# Supplementary figures and images for: Impeding Biofilm-Forming Mediated Methicillin-Resistant Staphylococcus aureus and Virulence Genes Using a Biosynthesized Silver Nanoparticles–Antibiotic Combination
Source: Biomolecules. 2025 Feb 11;15(2):266. doi: 10.3390/biom15020266 (PMC11852608; doi:10.3390/biom15020266)

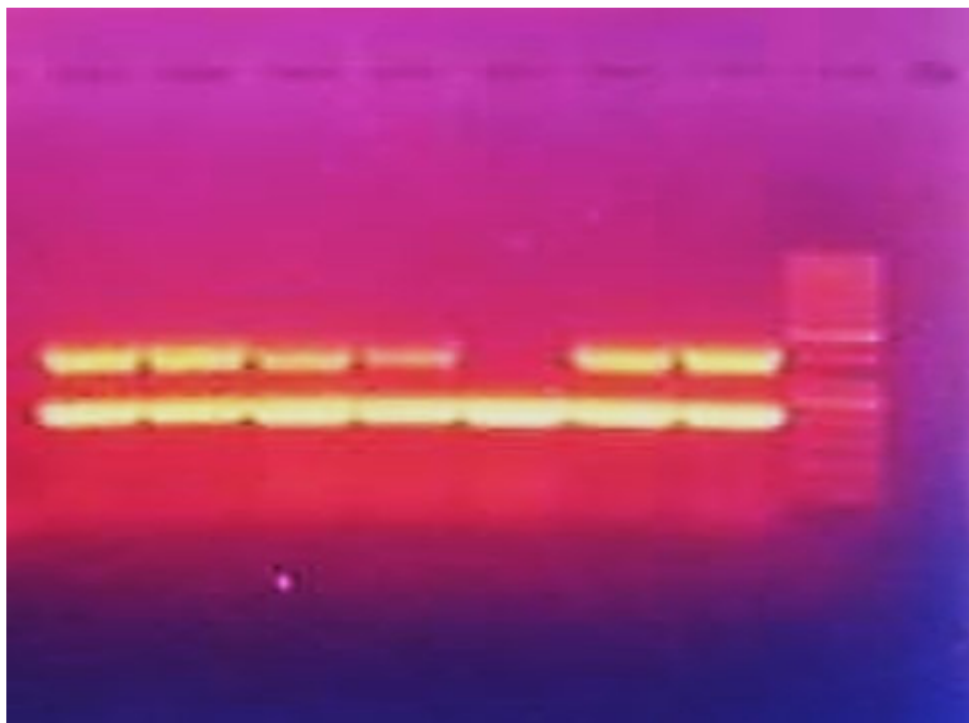

**Figure S1.** Original gel electrophoresis images of *icaA* and *icaD* genes.

Supplement: Supplementary file 1 [file biomolecules-15-00266-s001.zip › biomolecules-3418493-supplementary.pdf]
